# Shape Effect of Electrochemical Chloride Extraction in Structural Reinforced Concrete Elements Using a New Cement-Based Anodic System

**DOI:** 10.3390/ma8062901

**Published:** 2015-05-26

**Authors:** Jesús Carmona, Miguel-Ángel Climent, Carlos Antón, Guillem de Vera, Pedro Garcés

**Affiliations:** Civil Engineering Department, Universidad de Alicante, Ctra. San Vicente s/n, San Vicente del Raspeig 03690, Spain; E-Mails: jcarmona@ua.es (J.C.); ma.climent@ua.es (M.-A.C.); c.anton@ua.es (C.A.); guillem.vera@ua.es (G.V.)

**Keywords:** electrochemical chloride extraction, cement-based anodic system, shape effect

## Abstract

This article shows the research carried out by the authors focused on how the shape of structural reinforced concrete elements treated with electrochemical chloride extraction can affect the efficiency of this process. Assuming the current use of different anode systems, the present study considers the comparison of results between conventional anodes based on Ti-RuO_2_ wire mesh and a cement-based anodic system such as a paste of graphite-cement. Reinforced concrete elements of a meter length were molded to serve as laboratory specimens, to closely represent authentic structural supports, with circular and rectangular sections. Results confirm almost equal performances for both types of anode systems when electrochemical chloride extraction is applied to isotropic structural elements. In the case of anisotropic ones, such as rectangular sections with no uniformly distributed rebar, differences in electrical flow density were detected during the treatment. Those differences were more extreme for Ti-RuO_2_ mesh anode system. This particular shape effect is evidenced by obtaining the efficiencies of electrochemical chloride extraction in different points of specimens.

## 1. Introduction

Electrochemical chloride extraction (ECE) is nowadays considered an appropriate technique as a means of extending the service life of reinforced concrete structures. This method allows reducing the corrosion rate of steel rebar caused by chloride ions (Cl^−^). It basically consists of a migration of Cl^−^ ions from the reinforcement outward to the structure surface, thereby preventing its corrosive effect on steel. The removal of chloride ions is produced by applying a DC current between an anode in contact with an electrolyte, which is located on the outer surface of the concrete structure being treated, and its own rebar playing the role of cathode in this electrolytic process. Current density is in the range of 0.5–5.0 A/m^2^ relative to the exposed concrete surface, and the application time lasts a few weeks. This technique has been studied and developed since the 1970s [1,2,3], and thereafter the effectiveness of ECE has been solidly founded on a wide and rigorous series of research works [4,5,6,7]. An essential milestone in the development of this application was the registration of a patent named “Norcure” by Vennesland and Opsahl*.* [8], where the anode system was based on a Ti-RuO_2_ mesh. Afterwards, new studies were carried out aimed at improving some application conditions in order to simplify procedures and reduce costs, especially in the anode system. For this purpose, the use of multifunctional materials was considered, in particular cement-based conductive materials. Concrete, mortars and pastes are poor electricity conducting cement-based materials. However, their conductivity can be substantially enhanced by the addition of conductive materials such as carbon fibers or graphite powder. This procedure has been applied during recent years in order to provide new physical and chemical properties to cement-based materials [9,10,11,12,13,14,15]. One application has been the production of anodic systems for electrochemical treatments, such as cathodic protection [16,17,18,19]. Also, recent studies have focused on the development of cement-based anodic systems for ECE applications. The authors of this paper as well as others have recently carried out an investigation line using a conductive cement paste as anode for ECE treatments [20,21]. These studies have sufficiently proved that the efficiency of ECE when it is applied with an anode composed by a paste of graphite powder and Portland cement is similar to the one obtained with a reference anode (Ti-RuO_2_ mesh). The present study is a continuation thereof. Once the efficiency of this technique was demonstrated, the new objective was to check the characteristics of the electric transmission through this kind of anode. The shape and density of the electric flow caused by the direct current from the cathode (reinforced steel bars) to the anode were already cited by Tritthart [4]. Also, the electric flow of ECE was treated in an indirect way regarding the ECE capability to extract Cl^−^, according to the rebar arrangement of the treated element by Hope *et al.* [22] and Ihekwaba *et al.* [23]. More recently, the relationship between the rebar arrangement of structural elements and the ECE efficiency was studied by Garcés *et al.* [24]. All previously cited researchers have verified different particularities of this effect, but only in a qualitative way. In consequence, the objective of the present study consists in the evaluation of the electric flow structure produced during ECE applications with different types of anode systems in a quantitative way. It was based on checking the different efficiencies of ECE among core samplings extracted from several points of the different shaped specimens after the application of this electrochemical process.

## 2. Experimental Program and Materials

### 2.1. Case Studies Carried out

The research was carried out by applying ECE to four specimens of reinforced concrete, with the followings features:

Case study 1 application of ECE to a cylindrical specimen with Ti-RuO_2_ mesh anode.
Case study 2 application of ECE to a cylindrical specimen with graphite-cement paste anode.
Case study 3 application of ECE to a rectangular section specimen with Ti-RuO_2_ mesh anode. ECE efficiency is obtained for a core sample extracted on the center of the biggest face of the specimen.
Case study 4 application of ECE to a rectangular section specimen with Ti-RuO_2_ mesh anode. ECE efficiency is obtained for a core sample extracted on the cover zone of the rebar, just over the steel.
Case study 5 application of ECE to a rectangular section specimen with graphite-cement paste anode. ECE efficiency is obtained for a core sample extracted on the center of the biggest face of the specimen.
Case study 6 application of ECE to a rectangular section specimen with graphite-cement paste anode. ECE efficiency is obtained for a core sample extracted on the cover zone of the rebar, just over the steel.

To obtain the initial content of Cl^−^, one specimen of each shape was also prepared with the same procedures and materials, but without ECE application. Both were called the reference specimens.

### 2.2. Materials Used

#### 2.2.1. Concrete

In the present research, the electric flow density produced during ECE application in real structural elements is the main consideration, and the way to assess this density is to obtain the resultant ECE efficiency. For this approach, shape and size of the laboratory specimens are crucial. Therefore, these elements must be bigger than usual. They were molded as structural support-shaped, 1 m length, three of them in cylindrical shape with circular section 200 mm diameter, and another three as prisms, with rectangular section 200 × 300 mm. The cylindrical ones were reinforced with 6 longitudinal rebar 8 mm diameter, hexagonally arranged, including 3 stirrups 6 mm diameter uniformly distributed. Regarding the prismatic-shaped, the reinforcement was composed of 4 rebar 16 mm diameter located in the corners and 4 stirrups 8 mm diameter. In all cases, concrete cover was 40 mm thick. Concrete had a composition as is shown in Table 1. Water-cement ratio (w/c) was 0.6, higher than usual in construction. The reason is to obtain a porous concrete, in order to make easier the ionic transport across the concrete mass, evidencing in a clearer way the shape effect of the structural element during ECE application. In order to simulate a serious Cl^−^ contamination, 3.3% NaCl was added to the mixing water. Thus, the concrete contained 2% of Cl^−^ relative to cement mass.

**Table 1:** Dosage of the concrete for laboratory specimens.

Material	Dosage
Portland cement	350 kg/m^3^
w/c Ratio	0.6
Distilled water	210 kg/m^3^
Limestone aggregate 4/6	466 kg/m^3^
Limestone aggregate 6/12	679 kg/m^3^
Limestone sand	630 kg/m^3^
NaCl	3.3% (2% Cl^−^ relative to cement mass)

Both water/cement ratio and maximum content of Cl^−^ parameters were beyond the acceptable ranges in Spanish Code on Structural Concrete [25].

With the above-mentioned conditions, concrete reached a compression strength of 17.8 N/mm^2^ (UNE EN 12390-3:2009), a porosity of 17.0% (UNE 83980:2014) and a bulk density of 2.16 T/m^3^ (UNE EN 12390-7:2009).

#### 2.2.2. Anodic Systems

Reference anodic system consists of a mesh of braided wire 1 mm thick of titanium with ruthenium oxide. This wire is braided in diamond shapes 33 mm per 12 mm of diagonal length. The mesh was firmly attached covering the whole vertical surface of the specimens, between two layers of absorbent synthetic fabric to keep moisture. The electrical resistance of this mesh per unit length (1 m length × 1.2 m width) is 0.041 Ω/m. The mesh is connected to the positive pole of the electric source by a copper wire.

On the other hand, the conductive graphite-cement paste (GCP) is a homogeneous mixture of graphite powder, Portland cement and distilled water as is shown in Table 2. This product is applied by spraying with a compressed air gun on the vertical specimen surfaces forming a 2 mm thick overlay. The high water-to-solid ratio (w/s = 0.8) meets the fluidity requirements of the spraying application system. Dosage and thickness of GCP is adopted as a consequence of the good performances shown by this anode system in recent researches by the same authors [20,21].

**Table 2:** Dosage of graphite-cement paste for 5 kg of paste.

Material	Dosage (for 5 kg of paste)
Portland cement	1.389 kg
Graphite powder	1.389 kg
Distilled water	2.222 kg

#### 2.2.3. Electrolyte and Way of Moistening

The electrolyte for all the treatments was tap water, which was provided by a system able to maintain constant humidity during ECE process. That system consisted of a water pump and several pipes with droppers, assembled around the surface of each specimen. This constant drip irrigation system assures the presence of the electrolyte during the electrochemical procedure.

#### 2.2.4. Electric Power Source

The power source provided a direct current density, which was set in the range of 2–5 A/m^2^ relative to the exposed surface of the specimen. ECE treatments were designed with an electric charge density of 5 × 10^6^ C/m^2^, also relative to the exposed surface of the specimen. The voltage is therefore consequence of the constant current density and the resistance of the system. Thus, this voltage tends to raise as the ECE process progresses, since the resistivity of the specimens increases. In order to avoid an excessive increase, voltage must be monitored and controlled throughout the treatment. To control the voltage evolution and any circumstances during the whole research, specimens in treatment were remotely controlled by a WIFI IP camera. The European Standard CEN/TS 14038-2 proposes not exceeding the level of 40 V, for safety reasons as well as to prevent damages in the anode [26].

### 2.3. Assembly of Specimens

#### 2.3.1. Specimens with Ti-RuO_2_ Mesh Anode System

**Figure 1:**
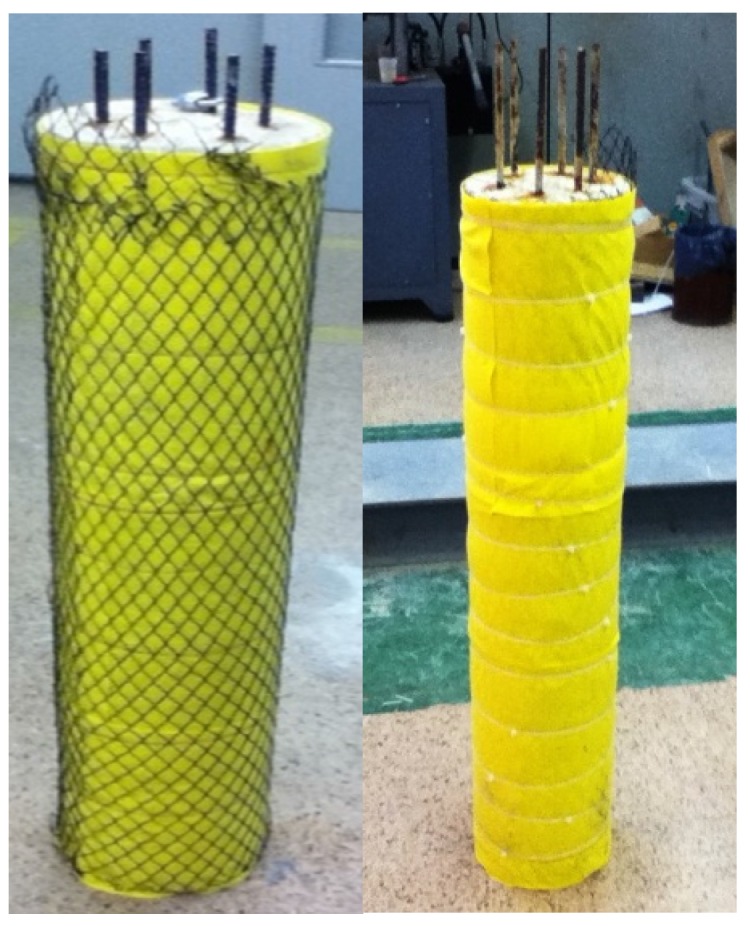
Anodic system composed by a Ti-RuO_2_ mesh embedded between two absorbent polymeric layers, wrapping concrete specimen.

One of each of the cylindrical and prismatic specimens was equipped with a conventional anode composed of a Ti-RuO_2_ mesh, as described in Section 2.2.2. The system consisted of two absorbent polymeric layers housing the Ti-RuO_2_ mesh between them. Those three layers were successively and firmly set up covering the whole exposed concrete surface of specimens. In this way, the specimens were able to retain moisture and thus, to maintain the electrolyte action during ECE process (see Figure 1). Specimens assembled as mentioned were placed into a recipient with water in order to immerse the pump of drip irrigation system. As for electrical connection, a protrusion was made in an extreme of the mesh to connect with the positive pole of the current source, closing the anodic circuit.

#### 2.3.2. Specimens with Graphite-Cement Paste (GCP) Anode System

As far as the rest of specimens (one of each type), a layer of GCP was applied by spraying on all vertical surfaces thereof. The spray was made with a compressed air gun, as a shotcrete system, forming an overlay around 2 mm thick. Then, specimens were moist-cured for 10 days in a curing chamber of relative humidity >95%. Finally, the paste coating was covered by the same polymeric layer mentioned above, and the same irrigation system was also installed to ensure the presence of the electrolyte. Anodic connections were made through two interconnected graphite felt fabric strips, 4 cm wide, firmly attached to the GCP layer, and united in turn with the positive pole of the source.

All specimens closed its cathodic circuit by connecting a rebar to the negative pole of the current source, assuming that all rebar are interconnected by the stirrups.

### 2.4. ECE Applications

#### 2.4.1. Specimens with Ti-RuO_2_ Mesh Anode System

ECE was applied with a current density of 5 A/m^2^, and charge density was 5 × 10^6^ C/m^2^. Wetting remained constant. Voltage, which was continuously monitored, never reached 40 V.

#### 2.4.2. Specimens with Graphite-Cement Paste (GCP) Anode System

Voltage increasing was higher than in Study 1. The voltage evolution is perhaps the most notable difference between both studies, which implies higher increase of resistivity with GCP anode than with a conventional Ti-RuO_2_ mesh anode. In order to prevent values above 40 V, two means were used, namely:

- a reduction in current density, but never under 1 A/m^2^; and
- the inclusion of pauses along the treatment.

The only effect of this change is the increase of the process time to achieve the same charge density of 5 × 10^6^ C/m^2^. ECE efficiency is directly related with electric charge density, while the times of application or the current density does not affect thereto, as was stated by Elsener *et al.* [27] and Polder *et al.* [28], and more recently by Sánchez de Rojas *et al.* [11]. Therefore, the comparison of ECE efficiencies among the different specimens is perfectly plausible because charge density was 5 × 10^6^ C/m^2^ for all studies.

### 2.5. Extraction of Core Samples

**Figure 2:**
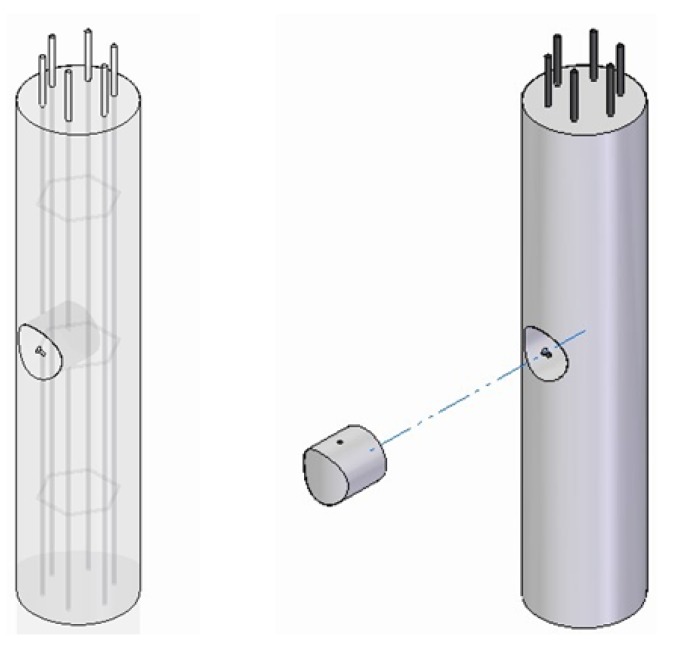
Extraction of core samples of cylindrical reinforced concrete specimens.

In order to work out the profiles of chloride content before and after ECE applications, different core samples were extracted from the specimens. All extracted cores were cylindrical 95 mm diameter and 40 mm depth, reaching just to the rebar location (see Figure 2).

From those cores, concrete dust samples were obtained by grinding each concrete core using a grinder device as recommended by RILEM TC 178-TMC [29]. This device is able to drill and pulverize accurately 2 mm of concrete each time with only turning the grinding crown 360°. Since the cores were 40 mm depth (concrete cover was 40 mm thick), 20 samples of concrete dust are obtained from each core, each sample belonging to every 2 mm thick layer. The subsequent chemical analysis will yield the chloride concentration at each depth.

Before carrying out the six studies, it was necessary to know the initial chloride content in the concrete specimens. To this end, a cross section was extracted, and the content of Cl^−^of both reference specimens was analyzed; one of each of the cylindrical and prismatic shape. These results were the initial chloride content profiles for the different studies carried out with each type of specimens.

**Figure 3:**
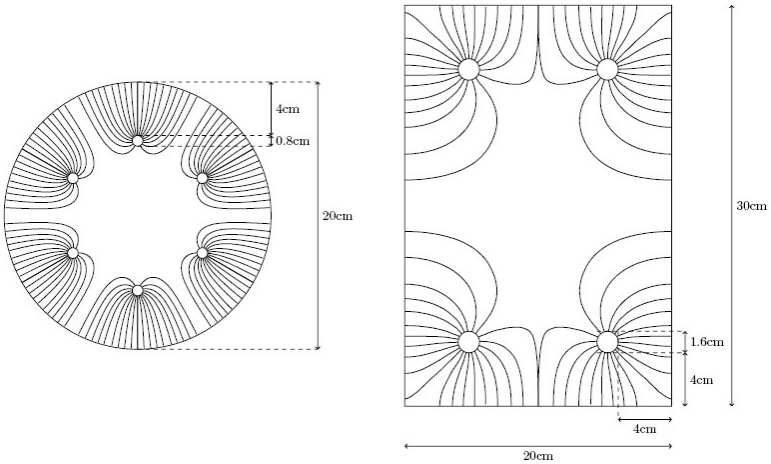
Different electric flow schemes during ECE application in an isotropic vertical element (circular section and regularly reinforced) and in an anisotropic vertical specimen (rectangular section and conventionally reinforced with rebar in the corners). Current streamlines, calculated using a standard finite element method, are shown.

Regarding the cylindrical ones, the extraction point was the central zone of the specimen, although the extraction efficiency should be independent of the sampling point. Indeed, in specimens of circular section with a regular rebar distribution, anode system is always equidistant from the cathode. The isotropy of this system implies that the electric field established during ECE treatment takes a uniform, or uniformly distributed, configuration (see Figure 3).

Nevertheless, geometry and heterogeneous rebar arrangements of rectangular section elements may produce differences in the electric flow created between the anode and cathode systems during ECE, as was mentioned in the Section 1. In 1998, Tritthart proposed his theory about electric flow produced during ECE process [4]. In the present study, this electric flow has been calculated through a standard finite element method. Results are shown in Figure 3.

**Figure 4:**
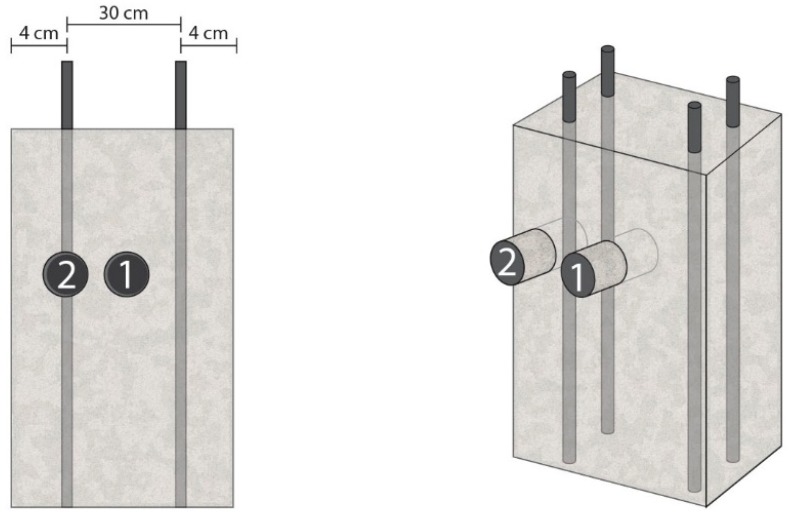
Extraction of two different core samples on prismatic reinforced concrete specimens. Point 1 is on the center of the biggest face of the specimen, and point 2 on the concrete cover, over the steel bar.

This electric flow configuration suggested that ECE efficiency must be different depending on the shape of the treated structural element. For the isotropic ones, it is evident that ECE efficiency is equal in whatever point of the element. On the contrary, ECE efficiency must vary along the same horizontal plane of an anisotropic treated element. This research tries to confirm and quantify the differences, and to evaluate the influence of the type of anode system in this topic. For this purpose, two different core samples were extracted from the rectangular shaped specimens (see Figure 4).

- Number 1 on the center of the biggest face of the specimen shown in Figure 4.
- Number 2 in the same horizontal plane, but on the concrete cover zone of the rebar (Figure 4).

### 2.6. Chloride Analysis

The determination of acid-soluble contents of the concrete samples was performed by potentiometric titration, according to the method stated by Climent *et al.* [30,31]. All Cl^−^ concentrations of concrete in this paper refer to acid-soluble chlorides, and are expressed as percentages, relative to cement mass. The amount of Cl^−^ contained in each sample, before and after ECE, allows plotting the different Cl^−^ profiles as shown in the figures of the Results and Discussion Section. The integration of the evolution of those profiles by the action of ECE represents the efficiency of the ECE treatments.

## 3. Results and Discussion

In this section, the set of results obtained during the related experiences is exposed. The objective is to test the effect of electrical flux lines produced during the application of ECE due to the anisotropy of structural elements, and how the use of different anode system could affect this consequence. To this end, the efficiency of ECE in different cases is obtained. On the one hand, considering the type of anode system (Ti-RuO_2_ or GCP), and, on the other hand, taking into account the different shape of treated specimens.

### 3.1. Study 1. Circular Section with Ti-RuO_2_ Mesh Based Anode System

**Figure 5:**
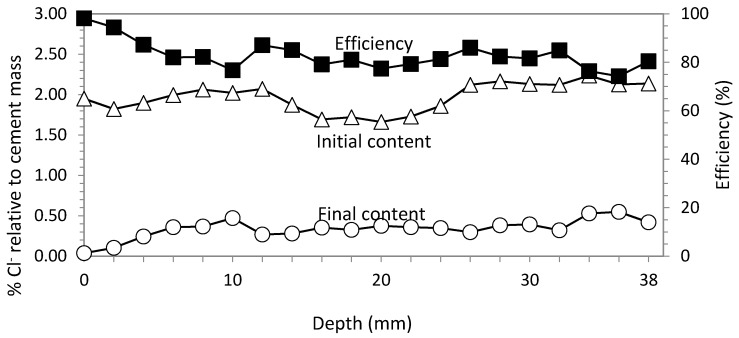
Study 1. Circular section specimen. ECE with Ti-RuO_2_ mesh anodic system. Current density 5 A/m^2^. Current charge density 5 × 10^6^ C/m^2^. Contents of Cl^−^ before and after ECE and differences in percentage relative to cement mass, which conform the efficiency profile.

Current density was 5 A/m^2^, and charge density 5 × 10^6^ C/m^2^. Wetting remained constant. Cl^−^ profiles before and after ECE treatment are represented in Figure 5. Also, the profile of differences between initial and final Cl^−^ content is plotted, showing the efficiency of ECE. The average of this parameter was 83.06%. Contents of Cl^−^ are always expressed as percentage relative to cement mass in concrete.

Voltage evolution during the process remains almost constant during the first five days, evolving upwards to achieve an increase of only 12 V at the end of treatment, and always below 40 V.

### 3.2. Study 2. Circular Section with GCP Based Anode System

In this second study, ECE is applied with the same electrical parameters as in Study 1, *i.e.*, current density 5 A/m^2^ and charge density 5 × 10^6^ C/m^2^. The anode system consists of a sprayed graphite-cement paste, as described in Section 2.3.2.

**Figure 6:**
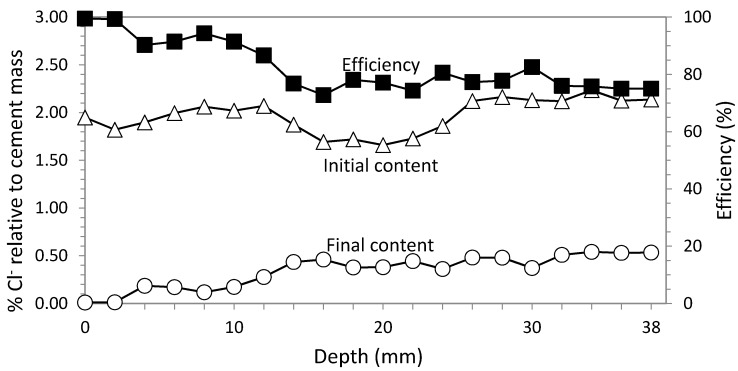
Study 2. Circular section specimens. ECE with GCP anodic system. Current density 5 A/m^2^. Current charge density 5 × 10^6^ C/m^2^. Contents of Cl^−^ before and after ECE and differences in percentage relative to cement mass, which conform the efficiency profile.

Figure 6 represents the Cl^−^ concentration profiles obtained before and after ECE application with a graphite-cement paste (GCP) anodic system, wetting constant. Extraction average of Cl^−^ was 82.60%.

In Study 2, the feeding voltage increase was higher than in Study 1. Pauses during the treatment were included to control the feeding voltage so that it remained below 40 V. Every time the voltage was near 40 V, a pause was applied. These pauses consisted of a current interruption of 24 h. When the current was restarted, a reduction of the feeding voltage in the range of 25%–30% of the last recorded value was found. For this study, three pauses were needed.

**Figure 7:**
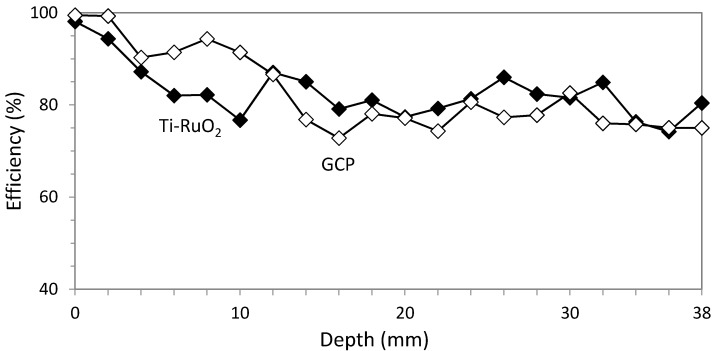
Comparative efficiency results for studies 1 and 2. Circular section specimens. ECE efficiency average with Ti-RuO_2_ mesh was 82.79% against 82.60% with GCP anodic system.

Efficiency results are very similar for ECE applied with both anode systems, as can be seen in Figure 7.

### 3.3. Rectangular Section with Ti-RuO_2_ Mesh Based Anode System

#### 3.3.1. Study 3. Core Sample in the Center of the Biggest Face

**Figure 8:**
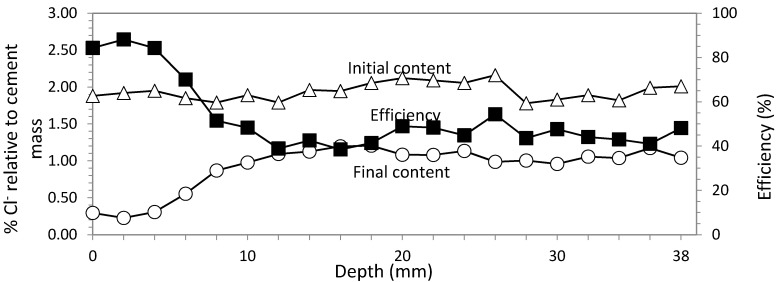
Study 3. Rectangular section specimens. ECE with Ti-RuO_2_ mesh anodic system. Variable current density between 3–5 A/m^2^. Current charge density 5 × 10^6^ C/m^2^. Contents of Cl^−^ before and after ECE and differences in percentage relative to cement mass, which conform the efficiency profile. Core sample extracted in the center of the biggest face.

As was said in Section 2.4, reinforced concrete specimens support type are also used for the second part of the laboratory tests, but in this case of rectangular section. Results of ECE efficiency for the core sample in the center of the biggest face (location 1 in Figure 3) are shown in Figure 8. Efficiency average for Study 3 was 52.52%.

In this case, the voltage evolution was faster than in circular section specimens. Consequently, around the middle of the treatment, the voltage was near 40 V. At that time, current density range was reduced from 5 to 3 A/m^2^ and the voltage dropped to 25 V. As is known, the only effect of this change is the increase of the process time to achieve the same charge density of 5 × 10^6^ C/m^2^.

#### 3.3.2. Study 4. Core Sample in the Concrete Cover Zone

**Figure 9:**
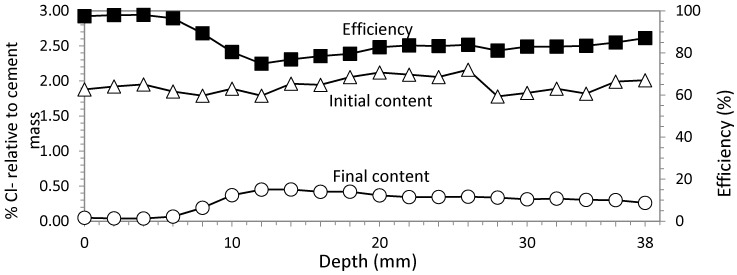
Study 4. Rectangular section specimens. ECE with Ti-RuO_2_ mesh anodic system. Variable current density between 3–5 A/m^2^. Current charge density 5 × 10^6^ C/m^2^. Contents of Cl^−^ before and after ECE and differences in percentage relative to cement mass, which conform the efficiency profile. Core sample extracted of the concrete cover up to the vertical rebar.

Nevertheless, when the core sample was extracted on the same horizontal plane, but on the concrete cover over the rebar (location 2 in Figure 3), chloride analysis shows an ECE efficiency almost double (see Figure 9). The average efficiency reached in this case 85.22%. Electric parameters (current density = 3–5 A/m^2^ and charge density = 5 × 10^6^ C/m^2^) were the same as in Study 3.

### 3.4. Rectangular Section with GCP Based Anode System

#### 3.4.1. Study 5. Core Sample in the Center of the Biggest Face

**Figure 10:**
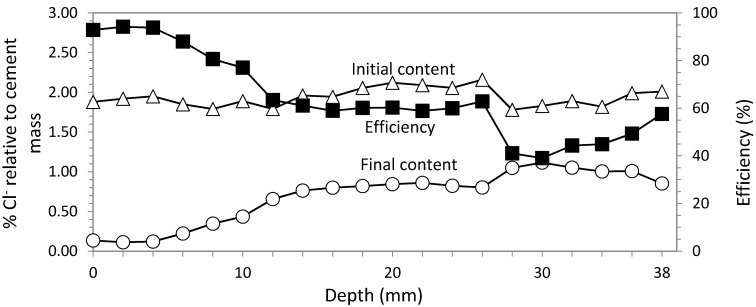
Study 5. Rectangular section specimens. ECE with GCP based anodic system. Variable current density between 2–3 A/m^2^. Current charge density 5 × 10^6^ C/m^2^. Contents of Cl^−^ before and after ECE and differences in percentage relative to cement mass, which conform the efficiency profile. Core sample extracted in the center of the biggest face.

This time, ECE was applied to rectangular section specimens using a GCP anode system as described before. The most significant difference consisted of a higher resistivity, manifested by a stronger increase of voltage during ECE process. Consequently, to maintain voltage below 40 V it was necessary to reduce current density to a range between 2–3 A/m^2^. Charge density was also 5 × 10^6^ C/m^2^ (see Figure 10).

The average efficiency was in this case 64.38%, a better value than in Study 3 (Ti-RuO_2_ mesh anode system—sample in the center of the biggest face).

#### 3.4.2. Study 6. Core Sample in the Concrete Cover Zone

**Figure 11:**
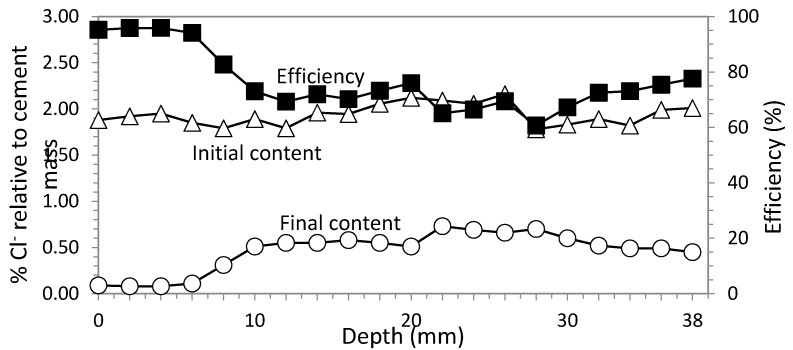
Study 6. Rectangular section specimens. ECE with GCP based anodic system. Variable current density between 2–3 A/m^2^. Current charge density 5 × 10^6^ C/m^2^. Contents of Cl^−^ before and after ECE and differences in percentage relative to cement mass, which conform the efficiency profile. Core sample extracted of the concrete cover up to the rebar.

In this last study, the core sample was extracted from the concrete cover zone, just over the rebar location. The values of the electric parameters were the same of Study 5: 2–3 A/m^2^ and 5 × 10^6^ C/m^2^. Initial and final Cl^−^ contents and efficiency are shown in Figure 11. The average efficiency in this case was 76.15%.

**Figure 12:**
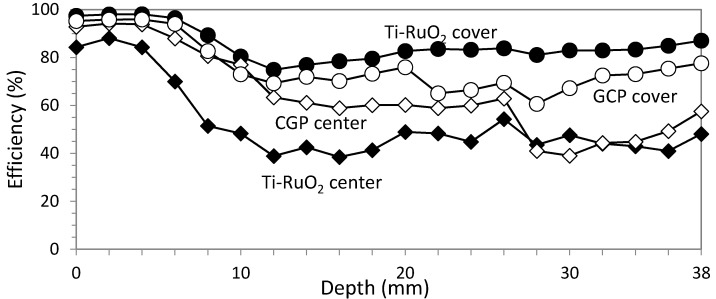
Comparative efficiency results for studies 3, 4, 5 and 6. Rectangular section specimens. ECE efficiency average with Ti-RuO_2_ mesh was 52.52% in the vertical axis of the largest side and 85.22% in the concrete cover, whereas for GCP, results were 64.38% and 76.15% for the same locations.

The different efficiencies obtained in studies 3, 4, 5 and 6 are shown in Figure 12, in order to compare them easier.

To summarize, results of efficiency averages of the six referred to studies are shown in Table 3.

**Table 3:** Summary of obtained efficiencies in the different studies carried out applying ECE to vertical shape specimens of reinforced concrete.

Study	Horizontal section shape of specimen	Anode system	Core sample location	Average efficiency (%)
1	Circular section	Ti-RuO_2_ mesh		82.79
2	Circular section	GCP		82.60
3	Rectangular section	Ti-RuO_2_ mesh	Center of the biggest face	52.52
4	Rectangular section	Ti-RuO_2_ mesh	Concrete cover over rebar	85.22
5	Rectangular section	GCP	Center of the biggest face	64.38
6	Rectangular section	GCP	Concrete cover over rebar	76.15

As for circular section specimens, ECE efficiencies show practically equal performance for both anode systems along the different depths and only 0.2% less efficiency average in GCP. The higher resistivity of GCP is also evidenced, but it is possible to pass the same electric charge density only by reducing current density or including some pauses in the treatment. This result confirms the feasibility of using a GCP anode system for ECE applications, as was also stated in previous research [20,21]. The high level of ECE efficiency, in either anode system and either sampling point, shows the uniformity and high density of electric flow lines produced between a regular cathode (rebars uniformly distributed) and an equidistant anodic surface, as was exposed in Section 2.5 and Figure 2.

Regarding specimens with rectangular sections and no uniformly distributed rebars (anisotropic element), ECE efficiency obtained in different points of the same horizontal section denoted the expected differences in the electric flow density at those positions, confirming the set out hypothesis. Indeed, the studies carried out with Ti-RuO_2_ mesh anode system gave significant difference of ECE efficiencies: 52.52% for the center of the biggest face *versus* 85.22% for the concrete cover over the rebar (62% more efficiency in the cover zone than in the center zone). This is consistent with the resultant electric flow density produced during ECE application to an anisotropic structural element, such as the prismatic specimens of this research (see Section 2.5 and Figure 2).

The same effect was detected in GCP anode system, but the difference of the electric flow density for the same horizontal section are significantly lower than in Ti-RuO_2_ mesh anode system (18% more efficiency in the cover zone than in the center of the biggest face). This effect would probably be related to the different shape of electric flow lines produced during ECE due to the fact that the contact between the Ti-RuO_2_ and the concrete surface is not perfect at all positions. As it is a metal mesh with certain rigidity and elasticity, the absolute adherence to the concrete surface is difficult to achieve. To confirm this question, subsequent studies are advisable. In any case, the fact that GCP is an overlay and its location as anode is the same structural element surface implies undoubtedly an advantage over other systems. Besides, efficiency averages among both extracted cores of each specimen after ECE application are also very similar, 68.87% for Ti-RuO_2_ mesh anode system and 70.26% for GCP anode system; 1.4% higher for GCP. All these qualities, coupled with the fact that the cost of the GCP materials is 8–10 times lower than that of the Ti-RuO_2_ mesh anode system, strengthen the option of GCP as anode system for ECE treatments.

## 4. Conclusions

The use in this research of specimens with a size close to natural scale allows determining the effect of electric flow density produced during ECE application to different shaped structural elements. Firstly, the measurement of the resultant percentage of removed Cl^−^ by the ECE action confirms the feasibility of achieving a similar ECE efficiency with a cement-based anode system such as GCP (a sprayed graphite-cement paste as overlay) to that obtained with the conventional Ti-RuO_2_ mesh anode system, whatever shape and arrangement of reinforced concrete elements to be treated.

As for the peculiar electric flow configuration produced by ECE application on anisotropic structural elements, GCP anode system shows similar effects to those of Ti-RuO_2_ mesh anode system, but with less extreme differences. This particular shape effect in the electric flow lines is deduced considering the different efficiencies of the treatments, which are significantly more marked with the conventional Ti-RuO_2_ mesh anode system. Further studies could be carried out to find out the reason of the obtained differences in ECE efficiency between both types of anodes.

It has also been noted that the shape and density of the electric flow as were calculated through a standard finite element method (see Figure 2) is directly related with the efficiency of ECE treatment. It is therefore probable to find the same effect in the rest of electrochemical applications to improve the service life of reinforced concrete structures, such as cathodic protection and realkalization. Thus, the results of this research suggest the advisability to move towards the design of isotropic structural elements for sustainability considerations.
